# Effect of gene polymorphisms on the levels of calcineurin inhibitors in Indian renal transplant recipients

**DOI:** 10.4103/0971-4065.70846

**Published:** 2010-07

**Authors:** T. Ashavaid, H. Raje, K. Shalia, B. Shah

**Affiliations:** Department of Lab Medicine and Research Laboratories, P. D. Hinduja National Hospital and Medical Research Center, Mumbai, India; 1 Research Department, P. D. Hinduja National Hospital and Medical Research Center, Mumbai, India; 2 Hurkisondas Nurrottmdas Hospital, Mumbai, India; 3 Lilavati Hospital, Mumbai, India

**Keywords:** Cyclosporine, CYP3A4-CYP3A5, gene polymorphisms, MDR-1, tacrolimus

## Abstract

The outcome of renal transplantation is improved by cyclosporine and tacrolimus. However, its success is limited by drug-induced nephrotoxicity. Therefore, monitoring their levels is important. These levels are influenced mainly by CYP3A4, CYP3A5 and MDR- 1 genes. These levels also affect target molecules of CNIs, mainly IL-2. Inter-individual differences in these levels have been attributed to SNPs in these genes and hence study of these SNPs assumes significance. So far no study has been carried out on Indian renal transplant recipients covering the SNPs of the genes involved in metabolism, efflux and drug target of CNIs, hence the data is lacking for Indian population. The aim is to study A-392G SNP of CYP3A4, A6986G SNP of CYP3A5, C3435T SNP of MDR-1 and T-330G SNP of IL-2 genes and correlate with CNI blood levels. Hundred healthy subjects and 100 consecutive renal transplant recipients; 56 on CsA and 44 on tacrolimus were genotyped by PCR followed by restriction enzyme assay for mentioned SNPs. No significant difference was observed between level/dose (L/D) ratio of CNIs and CYP3A4 and IL-2 SNPs. However, median L/D ratio for tacrolimus was significantly higher in subjects with CYP3A5*3/*3 (n = 24) (*P* = 0.011) and MDR- 1 3435TT (n = 18) (*P* = 0.0122). The findings from this study show that homozygous mutant patients for CYP3A5 and MDR-1 gene SNPs could be managed with lower tacrolimus dose to avoid nephrotoxicity.

## Introduction

Renal transplantation has been proved to be the optimal therapeutic option for ESRD (end stage renal d isease). The worldwide use of immunosuppressive drugs, especially calcineurin inhibitors (CNIs) i.e. cyclosporine A (CsA) and tacrolimus (tac or FK506); have enabled this treatment to obtain it’s current place. However, it is well recognized that responses to CNIs have significant inter- and intra-individual variation in transplant patients. These variations have considerable influence on the drug effects and human bodies. Overdose of these drugs has been proved to influence the lifespan of the allograft. These drugs also have their side effects; most serious of which is nephrotoxicity. Their narrow therapeutic indices, due to poor bioavailability and large inter-individual variability in drug administration, make the post-transplant therapeutic strategy difficult.

Many genetic and non-genetic factors such as organ function, drug interactions and the nature of the disease may influence the adverse effects of these drugs. Genetic factors are estimated to contribute much to the inter-individual variations in drug administration; it is estimated that genetics can account for 20 to 95 percent of variability in drug disposition and effects. Although many non-genetic factors, including age, organ function, concomitant therapy, drug interactions and the nature of the disease influence the effects of medications, there are numerous examples of cases in which inter-individual differences in drug response are due to sequence variants in the genes encoding drug-metabolizing enzymes, drug transporters or drug targets (Kalow *et al*. 1998,[1] Evans *et al*. 2003[2]).

Mainly CYP3A4 (Cytochrome P450 3A4) and CYP3A5 (Cytochrome P450 3A5) genes of cytochrome P450 gene family carry out the metabolism of CNIs in the liver to more than 30 metabolites and these metabolites are thrown out of the cell to the circulation for clearance by P-gp (Permeability glycoprotein) coded by MDR1 (Multidrug resistance-1) gene, IL-2 gene is the common target of CNIs to exert their immunosuppressive action. Polymorphisms in these genes have been associated with decreased metabolism, efflux and activity of CNIs respectively.

As Indian data regarding the association of the single nucleotide polymorphisms (SNPs) in these genes with the levels of the CNIs is lacking, we decided to study A-392G (CYP3A4), A6986G (CYP3A5), C3435T (MDR1) and T-330G (IL-2) polymorphisms in Indian renal transplant patients and to correlate their incidence with CNI blood levels. The hypothesis is that if the association exists for any of the polymorphisms studied with the increased CNI levels then the data could guide physicians to adjust the dose so as to avoid nephrotoxicity.

## Materials and Methods

The consecutive 100 renal transplant recipients receiving either CsA or tacrolimus (starting dose for CsA-8 mg/ kg/ day and for tacrolimus-0.15 mg/kg/ day) were included in the patient group. Blood sample collection was done in one 5 mL EDTA tube (The project was approved by the ethics committee of National Health Education Society, India, and consent was taken from each patent before taking sample). Samples were collected from our hospital (n = 12) as well as from Muljibhai Patel Urological Hospital (MPUH), Nadiad, Gujrat (n = 88). Transportation of the samples was done in a sealed container having ice pack. Control group comprised of consecutive 100 subjects having normal renal profile. Controls were selected to compare the genotype and allele frequencies of the polymorphisms included in the study for the patients and general healthy population. Control group was chosen from the subjects attending regular health check up program at our hospital. After the sample collection, blood samples of the patients as well as of the controls were stored at 4°C.

**Figure 1:**
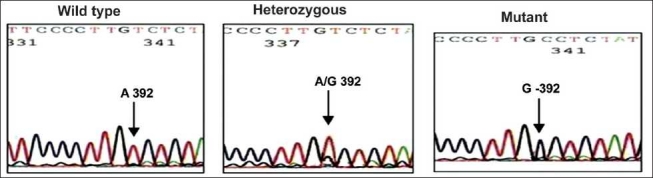
Sequencing results of CYP3A4 A-392G polymorphism

**Figure 2:**
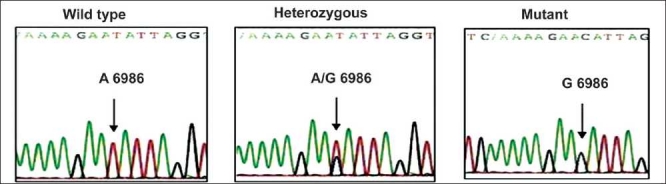
Sequencing results of CYP3A5 A6986G polymorphism

**Figure 3:**
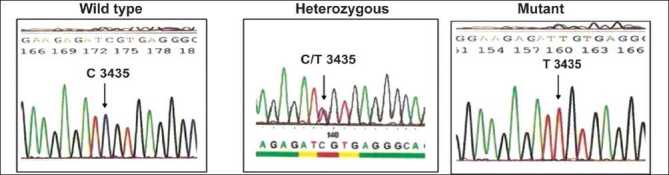
Sequencing results of MDR-1 C3435T polymorphism

**Figure 4:**
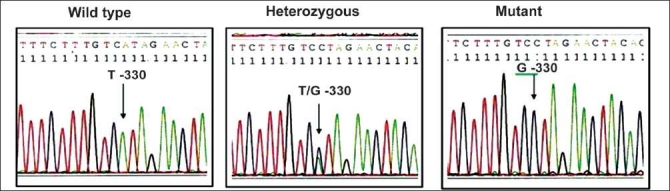
Sequencing results of IL-2 T-330G polymorphism

Collected blood samples were used for genomic DNA extraction by Miller *et al*.[3]. Extracted DNA from the samples was then subjected to DNA amplification by polymerase chain reaction (PCR).The genotyping of SNPs was performed as per the procedures described elsewhere.[4–7] For the confirmation of results obtained by restriction digestion method, representative samples were sent for automated sequencing by ABI prism to MWG Biotech, Banglore Genei or GenOmBio [Figures 1–4].

### Statistical analysis

Pearson’s Chi-Square test was applied to test the relationship of categorized dependant and independent variables. If the expected number in a Table was below 5 in any 2xn table, then Fisher’s exact test (one sided) was used as the test of significance. To study the effect on value, when classifying variables had greater than 2 subgroups, Kruskal-Wallis ANOVA (Analysis of Variance) was used as the test of significance. For all the tests *P* value < 0.05 was considered to be statistically significant. SPSS 15.0, STATA 10.0 and XLSTATS packages were used to perform the statistical analysis.

## Results

A total of 200 subjects, including 100 patients and 100 controls, were analyzed for the mentioned SNPs of CYP3A4, CYP3A5, MDR-1 and IL-2 genes. Among the consecutive 100 renal transplant patients included in the study, the incidence of kidney failure was observed more in the age group of 30-50 years. Gender wise distribution of patients shows that the prevalence of kidney failure was found more in males (80%) than in females (20%).

The correlation of SNPs was done with the sixth day post-transplant CNI levels. In order to segregate the patients who achieved higher sixth day CNI levels, both the categories of patients (i.e. those on CsA and on tacrolimus) were divided as per the therapeutic ranges of the respective drug levels.

In case of the patients receiving CsA (n = 56), the division was based on those who achieved < 1500 ng/mL 6^th^ day C2 level and those who achieved ≥ 1500 ng/ mL 6^th^ day C2 level. Likewise, in the case of tacrolimus receiving patients, the division was based on those who achieved < 10 ng/mL 6^th^ day trough level and those who achieved ≥ 10 ng/mL 6^th^ day trough level.

All the 56 patients receiving cyclosporine received azathiopurine and prednisolon along with it whereas, out of the 44 patients receiving tacrolimus, 27 received azathiopurine along with tacrolimus and 17 received mycophenolate mofetil along with it. On comparing the genotype frequencies of all the four polymorphisms studied between 100 healthy controls and 100 patients we did not find any significant difference [Table 1].

**Table 1:** Comparison of genotype frequencies between controls and patients

	Controls (n = 100)	Patients (n = 100)
CYP3A4		
AA	92	96
AG	6	3
GG	2	1
CYP3A5		
AA	6	3
AG	47	62
GG	47	35
MDR-1		
CC	12	14
CT	54	54
TT	34	32
IL-2		
TT	24	33
TG	53	54
GG	23	13

*P* = 1.000, NS (Fisher’s exact test); *P* = 0.0989, NS (Fisher’s exact test); *P* = 0.8462, NS (Chi-square test); *P* = 0.1354, NS (Chi-square test)

The comparison of genotype frequencies of all the four gene polymorphisms between the two subgroups of cyclosporine treated patients (those who achieved < 1500 ng/mL and those who achieved ≥ 1500 ng/mL 6^th^ day C2 levels) did not show any significant difference [Table 2].

**Table 2:** Comparison of genotype frequencies between the two groups of cyclosporine treated patients

	<1500 ng/ml	≥1500 ng/ml
CYP3A4		
AA	40	13
AG	2	0
GG	1	0
CYP3A5		
AA	2	0
AG	31	12
GG	10	1
MDR-1		
CC	12	0
CT	20	10
TT	11	3
IL-2		
TT	12	5
TG	25	7
GG	6	1

*P* = 1.000, NS (Fisher’s exact test); *P* = 0.357, NS (Fisher’s exact test); *P* = 0.107, NS (Fisher’s exact test); *P* = 1.000, NS (Fisher’s exact test)

However, on comparing the genotype frequencies of the studied SNPs between the two subgroups of tacrolimus treated patients (those who achieved < 10 ng/mL and those who achieved ≥ 10 ng/mL 6^th^ day trough levels) it was observed that the patients who achieved ≥ 10 ng/ mL 6^th^ day trough levels showed high prevalence of variant alleles in CYP3A5 and MDR1 genes polymorphisms [Table 3].

**Table 3:** Comparison of genotype frequencies between the two groups of tacrolimus treated patients and comparison of level/dose ratio of tacrolimus (Tac.) in the genotypes of CYP3A5 and MDR-1

	AA	AG	GG
CYP3A4			
<10 ng/ml	35	1	0
>10 ng/ml	8	0	0
CYP3A5	AA	AG	GG
<10 ng/ml	1	19	16
>10 ng/ml	0	0	8
MDR-1	CC	CT	TT
<10 ng/ml	2	23	11
>10 ng/ml	0	1	7
IL-2	TT	TG	GG
<10 ng/ml	13	19	4
>10 ng/ml	3	3	2
CYP3A5			
Tac. L/D ratio	AA	AG	GG
Median L/D ratio (ng/ml/mg/kg/day)	2.66	6.44	8.11
MDR-1			
Tac. L/D ratio	CC	CT	TT
Median L/D ratio (ng/ml/mg/kg/day)	6.22	6.22	8.83

*P* = 0.818, NS (Fisher’s exact test); *P* = 0.010, NS (Fisher’s exact test); *P* = 0.015, NS (Fisher’s exact test); *P* = 0.427, NS (Fisher’s exact test); *P* = 0.011, NS (Kruskal-Wallis ANOVA); *P* = 0.0122, NS (Kruskal-Wallis ANOVA)

## Discussion

The hypothesis of the present study states that if either of the mentioned polymorphisms was found to be associated with higher levels of either CsA or tacrolimus then it could guide physicians to adjust the dose of CNIs so as to avoid drug induced toxicity.

As CYP3A4 substantially contributes to the metabolism of many clinically important drugs, including CNIs, it was believed that the observed inter-individual difference in their metabolism is likely to be attributed to the polymorphic expression of this enzyme. However, the attempts to link SNPs in CYP3A4 gene with functional effects on drug pharmacokinetics have mostly shown negative results. Results from many studies have showed no significant pharmacological impact of this polymorphism on CsA pharmacokinetics. Von Ahsen *et al*. (2001)[8] have reported that this SNP had no significant effect on trough blood concentrations of CsA in 124 stable Caucasian renal transplant recipients. They also found no significant difference in the CsA doses needed to maintain similar drug levels in the patients with or without G-392 allele. Our study endorses these findings.

In the patient group, 56 patients who received cyclosporine did not show significant difference for G-392 allele frequency studied in the patients who developed <1500 ng/mL and ≥1500 ng/mL 6^th^ day C2 levels. Forty four patients who received tacrolimus also did not show significant difference in G-392 allele frequency studied in those who achieved <10 ng/mL and ≥10 ng/mL 6^th^ day trough levels [Table 3].

Several studies have shown that the dose of tacrolimus is associated with CYP3A5 SNPs (Zheng *et al*. 2003,[9] Zheng *et al*. 2004,[10] and Zhang *et al*. 2005[11]). In case of CsA, however, few studies have shown the role of this SNP on CsA pharmacokinetic characteristics. Hesselink *et al*.[12] found no association between CYP3A5 genetic polymorphism and dose adjusted predose concentration. Interestingly, unexpected results were reported in a study demonstrating that CsA oral clearance was significantly lower in CYP3A5 expressers than in the nonexpressers.[13] They suggested that this observation was artefact attributed to a linkage in their study population between CYP3A5 nonexpressers and MDR-1 3435T allele carriers. They failed to independently assess whether CYP3A5 correlated with any CsA pharmacokinetic parameter since all CYP3A5 nonexpressers were also MDR-1 3435T allele carriers.

Another report demonstrated that an effect of CYP3A5 intron 3 polymorphism was associated with CsA dosage to a lesser extent other than tacrolimus in stable renal transplant recipients.[14] Dose adjusted trough concentrations were three-fold and 1.6-fold higher in mutants (G/G) than in heterozygous (A/G) patients for tacrolimus and CsA, respectively.[12,15,16] Singh *et al*. 2009[17] have recently published a study on 73 North Indian patients on tacrolimus and have shown that CYP3A5 expressers were associated with significantly lower dose adjusted CsA/Tacrolimus concentrations and higher allograft rejection episodes in tacrolimus treated patients.

The findings from our study show that there is no significant difference in the CYP3A5*3 (G6986) allele frequency studied in the two groups of cyclosporine treated patients [Table 2]. However, for tacrolimus treated patients CYP3A5*3 allele frequency was significantly different in those who achieved <10 ng/ mL and ≥ 10 ng/mL 6^th^ day trough levels. Also, significantly high tacr olimus level/dose (L/D) ratio was observed in the homozygous mutant (CYP3A5 *3/*3) cases [Table 3].

Both CsA and tacrolimus are the substrates for P-gp. Numerous studies have associated SNPs in the MDR-1 gene with dosage of CsA and tacrolimus in organ transplant recipients. However, conflicting results have been reported. Von Ahsen *et al*. (2001)[8] found no significant difference in CsA doses needed to maintain similar CsA trough concentrations in 124 stable Caucasian renal transplant recipients. Others found no evidence supporting a role of MDR-1 C3435T in tacrolimus dose or CsA dose adjusted predose concentrations.[12] Macphee *et al*.[18] reported only a weak association of MDR-1 C3435T with tacrolimus concentrations and hence dose. A study,[19] comparing allele frequencies of MDR-1 C3435T SNP in 105 healthy Chinese volunteers and 50 renal transplant recipients, shows that the frequencies do not differ in both these groups (*P* > 0.05). Akbas *et al*.[20] in their study on 92 Turkish renal transplant recipients state that tacrolimus daily doses were significantly lower in the patients with 3435TT genotype at one and six months after transplant.

The finding from our study did not show any correlation of mutant genotype with higher cyclosporine levels. However, there was a significant correlation of 3435T allele with higher trough level of tacrolimus. Also, significantly high tacrolimus level/dose (L/D) ratio was observed in the homozygous mutant (T/T) cases [Table 3].

Polymorphisms in the promoter region of cytokine genes have been shown to influence their levels as well as some of them have been associated with toxicity or rejection episodes in case of solid organ transplant patients. Morgun *et al*. (2003)[21] have studied 67 heart and 63 renal transplant recipients for this SNP and found that wild type genotype (TT) is associated with kidney rejection. However, in the same study, no association was observed for heart transplant recipients. Holweg *et al*. (2003)[22] have also found no association of this SNP with rejection episodes in 301 heart transplant recipients. Our findings regarding the IL-2 T-330G polymorphism are at par with these results [Table 3].

To conclude, we have observed significant association of CYP3A5 A6986G and MDR1 C3435T polymorphisms with increased levels of tacrolimus in out study on 100 patients. Larger sample size can only strengthen this association. This data would be helpful to physicians so that by knowing the genotype of the patient before undergoing transplantation they would be able to decide upon the starting dose of tacrolimus so as to avoid high trough levels and consequently nephrotoxicity.
